# Synergistic Catalytic Effect of Ag and MgO Nanoparticles Supported on Defective BN Surface in CO Oxidation Reaction

**DOI:** 10.3390/ma16020470

**Published:** 2023-01-04

**Authors:** Anton S. Konopatsky, Denis V. Leybo, Vladislava V. Kalinina, Igor L. Zilberberg, Liubov Yu. Antipina, Pavel B. Sorokin, Dmitry V. Shtansky

**Affiliations:** 1 Research Laboratory Inorganic Nanomaterials, National University of Science and Technology “MISiS”, Leninsky Prospect 4, 119049 Moscow, Russia; 2 Institute of Solid State Chemistry and Mechanochemistry SB RAS, Kutateladze 18, 630128 Novosibirsk, Russia

**Keywords:** catalysts, hexagonal BN, nanoparticles, defect engineering, doping, CO oxidation, DFT calculations

## Abstract

Micron-sized supports of catalytically active nanoparticles (NPs) can become a good alternative to nanocarriers if their structure is properly tuned. Here, we show that a combination of simple and easily scalable methods, such as defect engineering and polyol synthesis, makes it possible to obtain Ag and MgO nanoparticles supported on defective hexagonal BN (*h*-BN) support with high catalytic activity in the CO oxidation reaction. High-temperature annealing in air of Mg-containing (<0.2 at.%) *h*-BN micropellets led to surface oxidation, the formation of hexagonal-shaped surface defects, and defect-related MgO NPs. The enhanced catalytic activity of Ag/MgO/*h*-BN materials is attributed to the synergistic effect of *h*-BN surface defects, ultrafine Ag and MgO NPs anchored at the defect edges, and MgO/Ag heterostructures. In addition, theoretical simulations show a shift in the electron density from metallic Ag towards MgO and the associated decrease in the negative charge of oxygen adsorbed on the Ag surface, which positively affects the catalytic activity of the Ag/MgO/*h*-BN material.

## 1. Introduction

Hexagonal boron nitride (*h*-BN) nanomaterials are a promising support for various metallic (Au, Ag, Pt, etc.) nanoparticles (NPs) used in heterogeneous catalysis [1]. These nanohybrids have shown their efficiency in the reactions of light alkene dehydrogenation [2], methane partial oxidation [3] and dry reforming [4], hydrogen peroxide generation [5], styrene production [6], and many others [7,8,9,10,11,12,13]. The *h*-BN edges can intensify chemical reactions by creating new active sites for the activation of molecules. In addition, B-OH bonds formed at the edges of defective *h*-BN serve as anchor centers for metal NPs [14]. However, readily available and inexpensive micron-sized BN powder, which could be used as a carrier for active NPs, lacks these advantages. There is a wide variety of defects that can be introduced into a material to improve its catalytic properties: single and double vacancies, adsorption atoms, dislocations, meso-, micro-, and macropores, edges, and interfaces [15,16]. Therefore, defect engineering and surface functionalization of economically attractive micron-sized BN materials is an important and promising scientific and technological approach.

Defect engineering includes several strategies: doping, chemical processing, and formation of active crystal edges and interfaces [17,18,19,20,21]. Various methods were used to create defects: ion implantation [22], high energy ball-milling [23], chemical etching [24], ultrasonic treatment [25], and oxidative annealing [26]. Annealing in an oxygen-containing atmosphere at moderate temperatures (500 °C) is an effective way to remove contaminants from the *h*-BN surface [27]. At temperatures of 850 °C and higher, this method can be used to form pores and pinholes in *h*-BN structures, with a thickness of up to four layers [28]. The observed trend is that the greater the number of layers, the higher the oxidation temperature required for defect formation. It is suggested that atomic oxygen adsorbed over the N atom in the 2D *h*-BN lattice tends to migrate further, while the O adatom adjacent to three B atoms saves its location [29]. This substitution of N atoms by O atoms determines the shape of the defects, which may have a linear or hexagonal morphology. Pits and holes in multilayer *h*-BN microparticles were also formed by oxidation etching of AgNPs [26], in which the oxidation process was additionally catalyzed by Ag, which led to the formation of defects of a certain shape and edges. At lower annealing temperatures, only the perimeter of the *h*-BN microparticle was etched. Transition metal oxides preliminary deposited on *h*-BN facilitates oxidative etching [30]. Thus, a controlled number of pores and active sites can be formed on the *h*-BN surface, which will increase the material activity with respect to the adsorption of gas molecules.

Another promising approach to the functionalization of catalytic supports is their doping with metal oxides. It has been shown that *h*-BN-based substrates promote efficient dispersion of V_2_O_5_-CuO-TiO_2_ oxides and reduce the catalyst deactivation rate [31]. The oxide composition, and, hence, its properties in the catalytic process, can be controlled through *h*-BN surface functionalization. For example, a decrease in the number of B-O surface bonds in the CoO/BN system promotes the formation of Co^0^ species contributing to catalyst activity [32]. The oxide catalytic activity can change significantly in heterostructures. For example, MgO is inactive in the CO oxidation reaction, but exhibits enhanced catalytic activity in the ultrathin MgO/Ag heterostructure and some other systems [33,34,35]. An increase in the rate of CO oxidation has been associated with the presence of AgNP/MgO interfaces. Theoretical calculations have shown that molecular oxygen can be activated over O-vacancies containing the MgO (100) plane decorated with AuCu clusters [36]. The introduction of Mg into the *h*-BN structure can be carried out during the synthesis. It has been shown that Mg^2+^ ions penetrate the *h*-BN crystal lattice, filling B vacancies and increasing oxygen adsorption [37]. A combined approach based on defect formation and doping with metal oxides is promising for improving the catalytic properties of *h*-BN-based catalysts, as was recently demonstrated for the MnO-CeO-BN system [38]. Note that the uniform distribution of catalytically active NPs is especially important for enhanced catalytic performance.

Among many catalytic processes, special attention is paid to the model reaction of carbon monoxide oxidation. Due to its relative simplicity, this reaction is often used for the analysis of various catalytic mechanisms [39,40,41]. However, CO oxidation is also of great practical importance in the development of sensors, gas masks, converters for the automotive industry, etc. [42]. Thus, the aim of this work is the formation of defects in *h*-BN microparticles with the simultaneous precipitation of MgO NPs on their surface by oxidative annealing and revealing their role in the CO oxidation reaction. To do this, *h*-BN micropellets containing trace amounts of Mg (<0.2 at.%) were subjected to high-temperature annealing to form surface defects and MgO NPs. The resulting MgO/*h*-BN material served as a substrate for the deposition of active Ag NPs. The high catalytic effect of Ag/MgO NPs in the CO oxidation reaction is explained by the synergistic effect of *h*-BN surface defects, ultrafine Ag and MgO NPs, and the higher electrophilicity of adsorbed oxygen at the Ag/MgO interface.

## 2. Materials and Methods

### 2.1. Experimental Details

**Figure 1:**
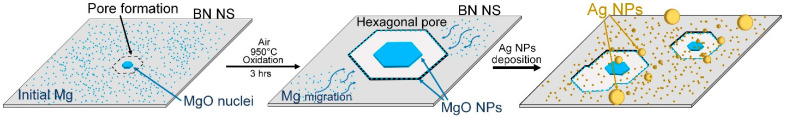
Scheme for obtaining Ag nanoparticles supported on BN samples with hexagonal-shaped surface defects and defect-related MgO nanoparticles.

A powder of *h*-BN micropellets (Plasmotherm, Russia) with a diameter of up to 10 μm and a thickness of ~300 nm (hereinafter referred to as BN_m_), obtained using a slight excess of Mg precursor, was annealed in air in a horizontal tube furnace (Nabertherm, Lilienthal, Germany) using an Al_2_O_3_ crucible at temperature 950 °C for 3 h. After annealing, the crucible was removed and cooled to room temperature. The resulting powder was designated as BN_a_. Both types of powders were used as substrates for the deposition of Ag NPs. The powder (200 mg) was dispersed in a synthesis medium (polyethylene glycol (PEG-400) with a molecular weight of 400) using an immersion sonotrode (Bandelin SONOPULS HD2000, Berlin, Germany) for 3 min. Then, 100 mg AgNO_3_ was added into the medium. Quartz glass with the resulting dispersion was placed under a UV lamp (40 W, λ = 253 nm) and subjected to UV irradiation for 20 min with constant stirring. After synthesis, the obtained heterostructures were repeatedly rinsed in distilled water by centrifugation (8000 rpm, 25 min) and dried in a fume hood overnight. The scheme for obtaining samples is shown in Figure 1.

The structure and morphology of the samples were studied using scanning electron microscopy (SEM) on a JEOL 7600F instrument (Tokyo, Japan) equipped with Oxford EDX analyzer (High Wycombe, UK) and scanning/transmission electron microscopy (SEM/TEM) on a FEI Osiris 200 kV microscope (Waltham, MA, USA) with an X-FEG electron source. The phase composition was characterized by X-ray diffraction (XRD) using a D2 Phaser diffractometer (Bruker, Billerica, MA, USA) using Cu-Kα radiation at λ = 1.54178 Å. The Raman spectra were obtained on a NTEGRA Spectra II setup with a red laser (632.8 nm) as the excitation source. Infrared absorbance spectra were recorded by Fourier-transform infrared spectroscopy (FTIR) using a Vertex 70v spectrometer (Bruker, Billerica, MA, USA). The surface chemical state was analyzed by X-ray photoelectron spectroscopy (XPS) on a PHI VersaProbe III (Kanagawa, Japan) device equipped with a dual charge neutralization system, multichannel detector, and a monochromatic microfocus scanning X-ray source (Al Kα, 1486 eV). The resulting XPS spectra were calibrated by setting the C1s peak at 285.0 eV. Specific surface area was determined by BET method on a surface area analyzer (Quantachrome Instruments, Graz, Austria) using nitrogen gas sorption. The experiments were carried out at a temperature of 77 K for 30 min.

The catalytic activity of the obtained materials in the carbon monoxide (CO) oxidation reaction was studied in a fixed-bed continuous-flow reactor at ambient pressure. The amount of 0.1 mL of catalyst (fraction 200 μm) diluted with quartz granules was placed in a stainless steel tube. Activity measurements were carried out in a gas flow of 2 nmL/min CO, 4 nmL/min O_2_, and 30 nmL/min He (VHSV = 21,600 h^−1^). The reaction products were analyzed on-line on a Crystal 5000 gas chromatograph (Yoshkar-Ola, Russia) equipped with thermal conductivity detectors, packed columns NaX for the analysis of O_2_ and CO, and Haesep N for the analysis of CO_2_.

### 2.2. Simulation Details

All calculations of the atomic structure and electronic properties of the considered phases were performed within the framework of generalized gradient approximation in the Perdew–Burke–Ernzerhof (GGA-PBE) parameterization [43], and the plane wave basis set was implemented in the VASP package [44,45,46,47]. The projector augmented wave (PAW) method [48] and the plane–wave cutoff energy of 500 eV with a Monkhorst–Pack [49] 3 × 3 × 1 k-point mesh were used. The Ag surface is modeled as a 4-layer slab. The Ag/MgO interface is simulated by connecting two 4-layer Ag slabs and MgO.

## 3. Results and Discussion

### 3.1. Mechanism of Defect Formation

**Figure 2:**
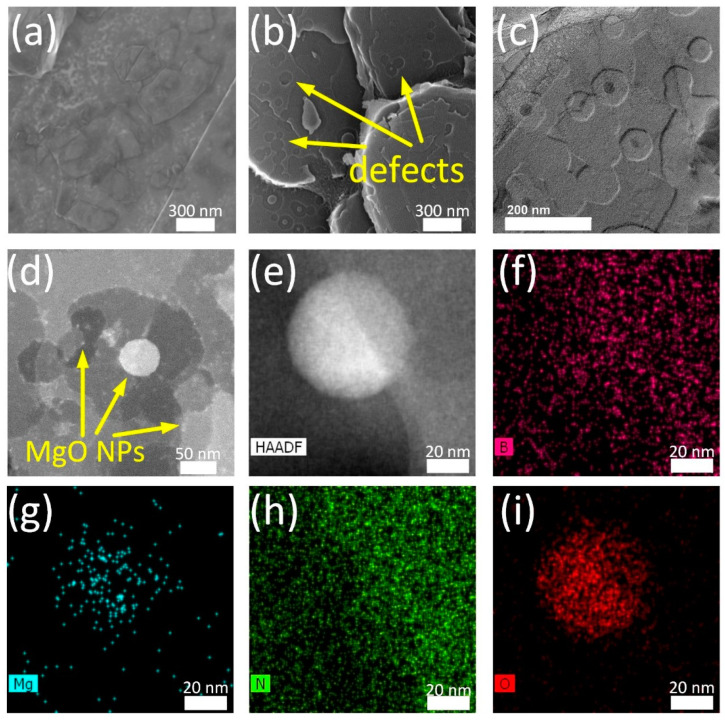
SEM (**a**,**b**), TEM (**c**), and HAADF STEM (**d**,**e**) images and corresponding EDXS elemental maps (**f**–**i**) of BN_m_ (**a**) and BN_a_ (**b**–**i**) samples.

After annealing, typical hexagon-shaped defects are observed on the BN surface, corresponding to the hexagonal structure of BN lattice, with a size of 50–90 nm and a depth of several atomic layers (Figure 2b–d). Quite often, several individual hexagonal defects merge into one larger irregularly shaped pore (Figure 2c,d). In the center of the pores, MgO particles, approximately 50 nm in size, also hexagonal shape, were often observed, as evidenced by the corresponding HAADF STEM image and EDXS elemental maps (Figure 2e–i). As they grow, the MgO particles acquire a spherical morphology. It is also interesting to note that the pore edges are decorated with smaller MgO nanoparticles (NPs), 2–5 nm (Figure 2d). It was reported that in the Mg-doped BN, Mg^2+^ ions occupy B vacancies [37], and Mg is bonded to nitrogen [50]. In oxidized BN, oxygen atoms substitute nitrogen atoms and form B-O bonds [51]. At high temperatures, due to the enhanced adatom mobility, the Mg and O atoms leave their positions in the BN lattice and form a MgO nucleus, which becomes the defect center. The reaction further proceeds by the addition of Mg and O atoms, the destruction of B-N bonds, and the retreat of the BN defect border from the MgO NP. The active edges of BN defects serve as anchor centers for nanometer-sized MgO NPs (Figure 2d). Note that irregular-shaped pits and small boron oxide particles were also observed during thermal oxidation of *h*-BN in ambient air, as reported elsewhere [26,52].

### 3.2. Structure and Surface Chemical State

**Figure 3:**
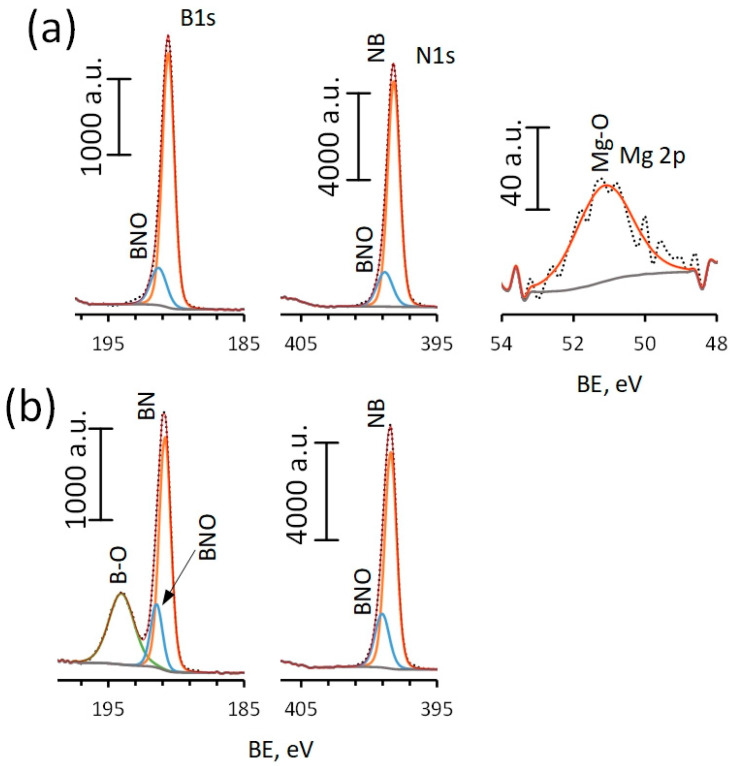
XPS spectra: BN_m_ (**a**) and BN_a_ (**b**) samples.

The XPS spectra of sample BN_m_ show single peaks at ~190.7 eV (B1s) and ~398.3 eV (N1s) (Figure 3a). Deconvolution of these peaks indicates the presence of B-N (as a main component) and BNO bonds. The position of the Mg 2p peak at ~51.1 eV indicates the presence of Mg^2+^ species [53]. It can be assumed that Mg is bound to oxygen either in the BN lattice or by forming MgO clusters on the surface. According to the results of XPS analysis, the Mg content in the surface is low (0.9 at.%). Analysis of the elemental and phase compositions shows that after annealing, the content of nitrogen decreases two times, and oxygen increases three times (Table 1). Boron becomes predominantly associated with oxygen in the form of oxide B_2_O_3_ (Figure 3b).

**Figure 4:**
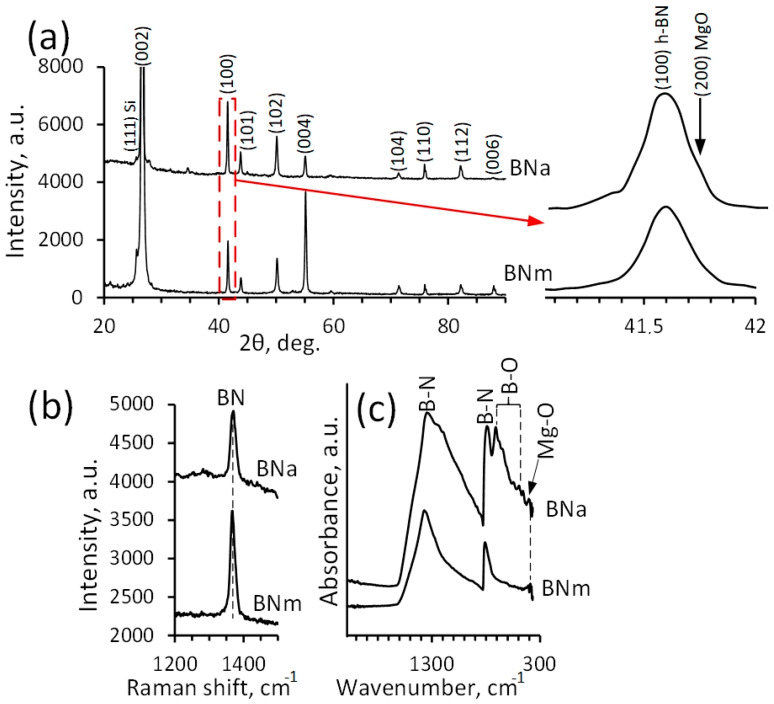
XRD patterns (**a**), Raman spectra (**b**), and FTIR spectra (**c**) of BN_m_ and BN_a_ samples.

Note that the oxidation process affects mainly the near-surface layer of the BN sample, while *h*-BN is retained as the main structural component in the bulk. No other peaks, except for BN and the Si substrate, are observed in the corresponding XRD patterns (Figure 4a). However, the BN (100) peak exhibits an additional shoulder on the large angle side, which indicates the contribution of MgO (200). The Raman spectra show maxima at 1340 cm^−1^ corresponding to the B-N vibrational modes (Figure 4b). In the BN_a_ sample, the FWHM value slightly increases, which may be due to the formation of defects. The FTIR spectra of BN samples before and after annealing are significantly different (Figure 4c). After annealing, an additional peak appeared at approximately 700 cm^−1^, and the main peak at 1300 cm^−1^ broadened significantly towards lower wavenumber values, which indicates the presence of B-O bonds. Small peaks observed in the FTIR spectra in the range of 400–450 cm^−1^ indicate stretching vibrations of the Mg–O bond (Figure 4c) [54]. BET analysis showed that the specific surface area of the pristine BN_m_ powder was 23 m^2^/g and slightly increased to 26 m^2^/g in the BN_a_ sample. Thus, the creation of surface defects did not significantly affect the specific surface area. This may be due to the competing sintering of BN nanoflakes during high-temperature annealing.

**Figure 5:**
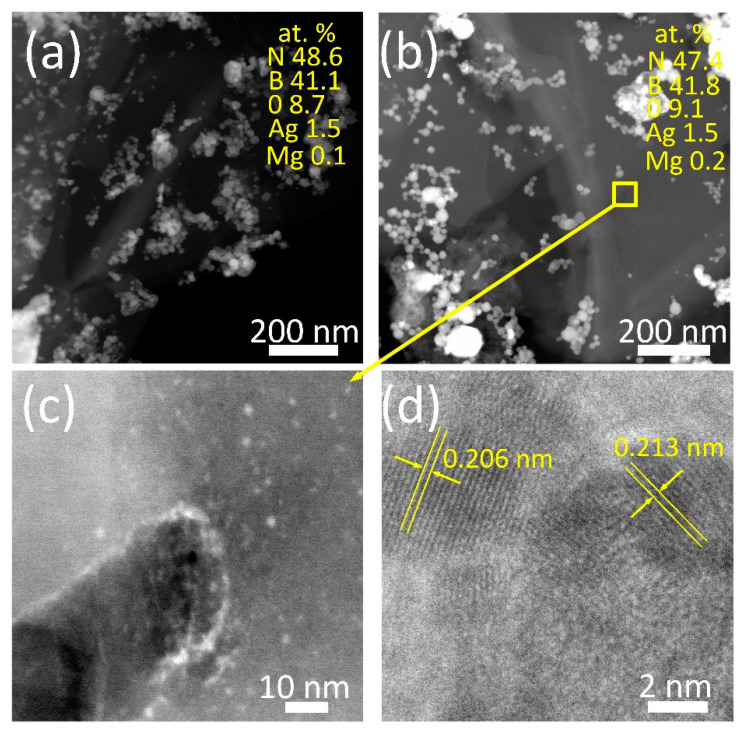
HAADF STEM (**a**–**c**) and high-resolution TEM (**d**) images of Ag/BN_m_ (**a**) and Ag/BN_a_ samples.

According to the EDXS analysis, the Ag content in both samples is the same (~1.5 at.%). The results of the structural analysis show that the BN surfaces are covered with Ag NPs having two characteristic sizes. Relatively large Ag NPs are 8–35 nm in size (Figure 5a,b). At higher magnification, very small Ag NPs (1–2 nm in dimension) are observed on the BN surface. In sample BN_a_, Ag NPs precipitated predominantly along the defect boundaries (Figure 5c). The high-resolution TEM image shows the coexistence of Ag and MgO NPs (Figure 5d). The observed characteristic interplanar spacings of 0.206 and 0.213 nm can be attributed to the (200) MgO (JCPDS card No. 89-7746) and (111) Ag (JCPDS card No. 04-0783) planes, respectively.

### 3.3. Catalytic Activity Tests

**Figure 6:**
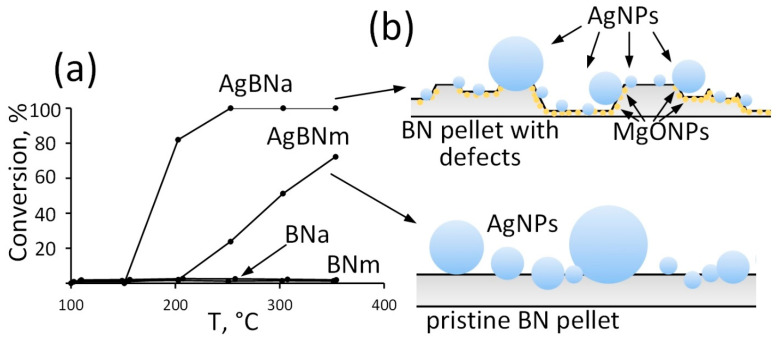
CO conversion (**a**) and schematic structure illustration (**b**).

The results of the catalytic activity tests are presented in Figure 6. Neither BN_m_ nor BN_a_ show a noticeable catalytic effect in the CO oxidation reaction. In contrast, sample AgBN_m_ exhibits a pronounced catalytic effect with an offset temperature of ~210 °C, and 75% conversion is reached at 300 °C (T_75_ = 300 °C). Sample AgBN_a_, with the reaction onset temperature of ~150 °C and complete CO conversion at 250 °C (T_100_ = 250 °C), has the highest catalytic activity. This may be associated with the formation of Ag/MgO interfaces, the positive effect of which on the CO oxidation has been demonstrated theoretically and experimentally [33]. It is also known that defects in the *h*-BN structure increase catalytic activity, including in CO oxidation [55,56]. It is assumed that the role of *h*-BN defects is to increase the energy of O_2_ adsorption and oxygen activation due to the weakening of the O-O bond. It should be noted that the catalytic activity of the obtained micron-sized BN-based catalyst is comparable to *h*-BN nanosheets supported Ag NPs [57,58].

### 3.4. Computational Modeling

**Figure 7:**
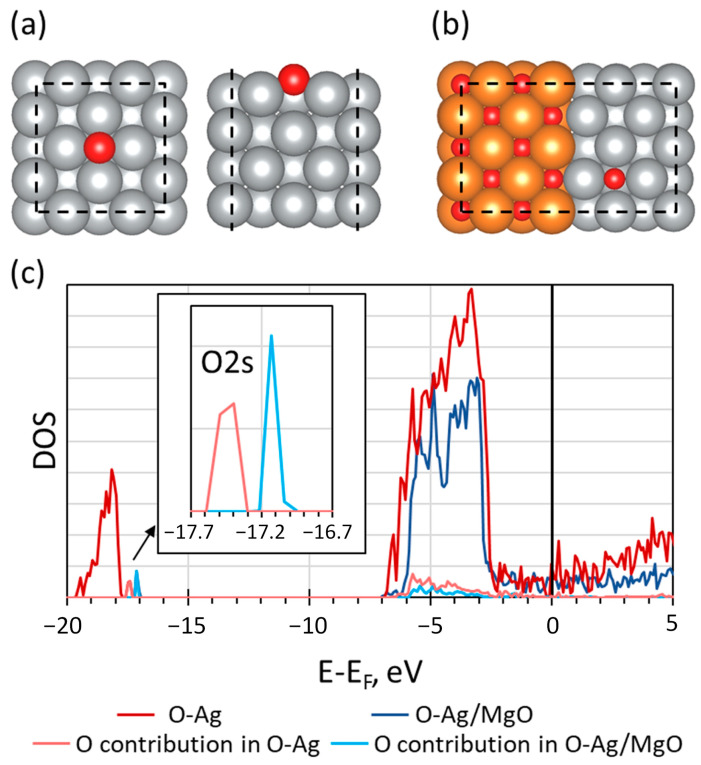
Atomic model of oxygen on the Ag surface (**a**) and Ag/MgO interface (**b**). O, Ag, and Mg are marked in red, gray, and orange, respectively. The unit cell is shown with dashed lines. Density of electronic states of oxygen adsorbed on Ag surface (red color) and Ag/MgO interface (blue color) (**c**). The partial density of oxygen electronic states is represented by pink and cyan colors for the first and second cases, respectively. The inset shows the enlarged region of O2s levels. The Fermi level is not shown.

To reveal the effect of MgO on the catalytic activity of the surface Ag NPs, the following model was developed. It is known that Ag actively participates in selective oxidation processes, forming the so-called electrophilic oxygen species [59]. This activity is fingerprinted by the binding energy (BE) of the deep level, which can be measured using XPS: the higher the BE, the higher the activity. This is because a higher BE indicates the presence of oxygen with a lower negative charge. Such surface oxygen is electrophilic and should be active in oxidative catalysis. We theoretically studied this effect by analyzing the deep oxygen 2s level (O2s). Since there are no XPS data for these systems in the literature, the problem can be qualitatively solved by comparing the O2s density of states (DOS) for oxygen on the pure Ag surface (Figure 7a) and at the Ag/MgO interface (Figure 7b).

Calculations show that the position of the O2s level maximum relative to the Fermi level is −17.1 and −17.5 eV for O-Ag and O-Ag/MgO, respectively. This means that in the presence of Mg-O, the adsorbed oxygen atom is more electrophilic than in the case of pure Ag. This effect can be qualitatively explained by a shift in the electron density from metal Ag towards Mg-O and a subsequent decrease in the negative charge of oxygen adsorbed on the Ag surface. Therefore, one can assume a higher catalytic activity of the Ag-MgO heterostructure.

## 4. Conclusions

A simple and easily scalable method of Ag/MgO/*h*-BN catalyst fabrication was developed. Hexagonal-shaped surface defects were formed in Mg-containing *h*-BN micropellets via oxidative annealing. MgO nanoparticles (NPs) formed during this step and occupied the pore center as well as defect edges. Defective, MgO-decorated BN powder was used as a substrate for AgNPs deposition by polyol method. The Ag/MgO/*h*-BN catalyst showed a significantly higher catalytic activity in the CO oxidation reaction compared with defect-free *h*-BN-supported Ag NPs. Theoretical analysis suggests that the enhanced catalytic activity is due to the higher electrophilicity of adsorbed oxygen at the Ag/MgO interface compared with the pure Ag surface.

## Figures and Tables

**Table 1:** Surface chemical state before and after annealing.

Sample	B1s			N1s		Elemental Composition, at. %		
	BN	BNO	B_2_O_3_	BN	BNO	B	N	O
BN_m_	83.5	16.5	0	83.4	16.6	48.7	40.1	10.3
BN_a_	52.1	14.1	33.8	77.2	22.8	41.2	22.6	36.2

## Data Availability

Not applicable.
